# Supplementary figures and images for: Listeria monocytogenes infection rewires host metabolism with regulatory input from type I interferons
Source: PLoS Pathog. 2021 Jul 8;17(7):e1009697. doi: 10.1371/journal.ppat.1009697 (PMC8266069; doi:10.1371/journal.ppat.1009697)

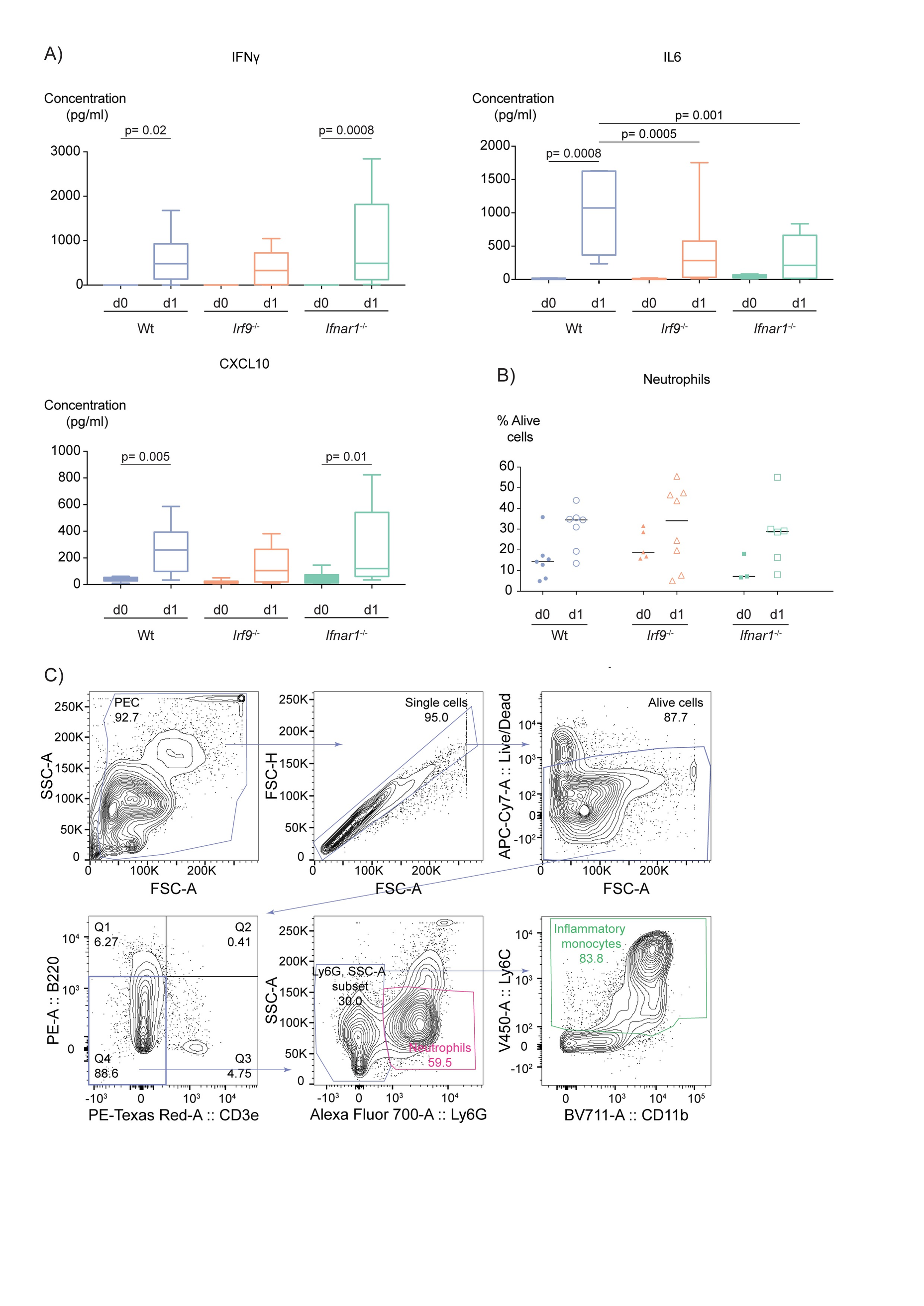

Supplement: S1 Fig — (A) Cytokine levels of mice uninfected or infected for one-day. (B) Neutrophil recruitment into the peritoneal cavity of PBS-treated mice or one day post-infection. (C) Gating strategy for flow cytometry of PECs in Figs 1 and S1. P values were calculated with two-way ANOVA test. (TIF) [file ppat.1009697.s001.tif]

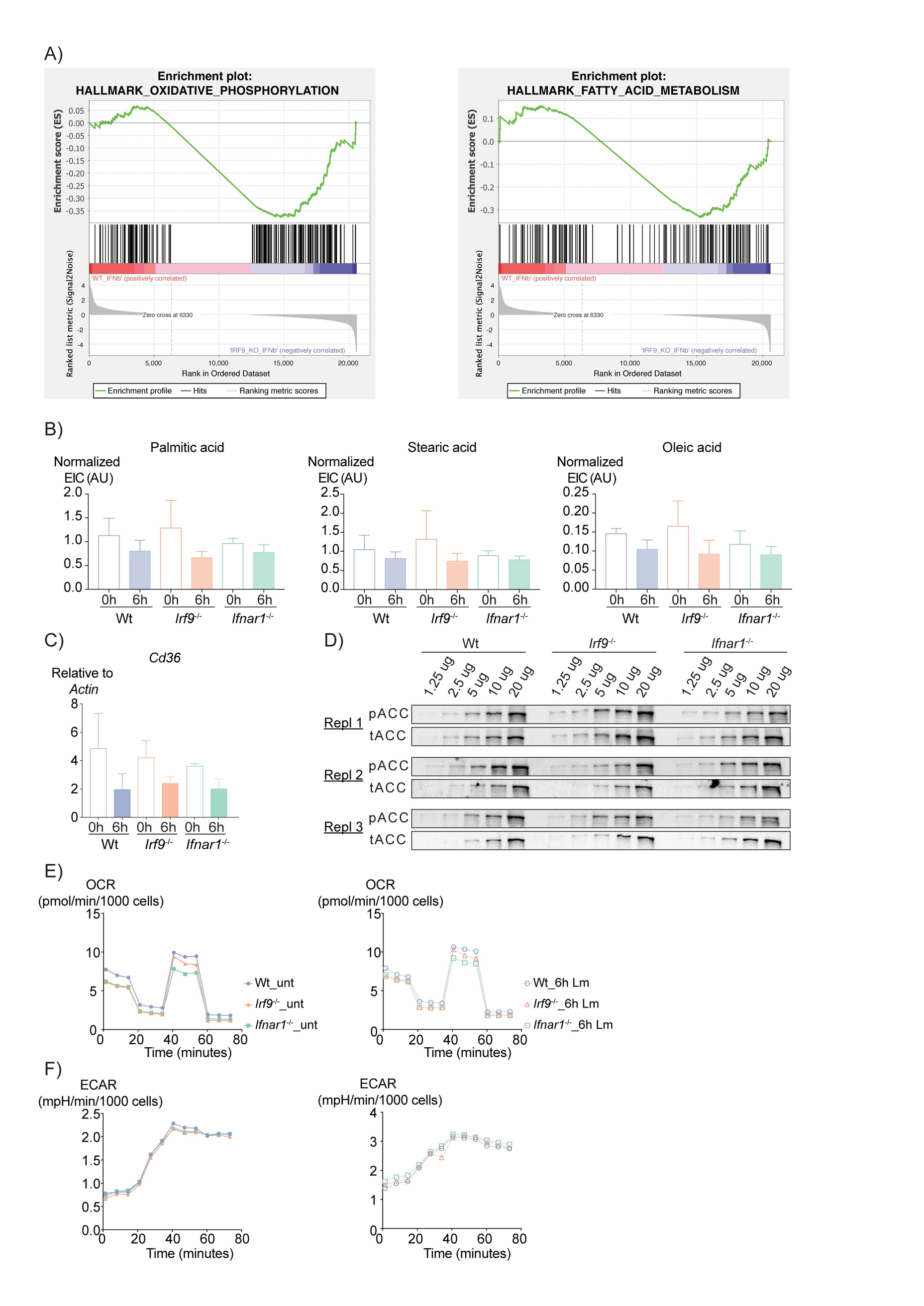

Supplement: S2 Fig — (A) GSEA enrichment plots for OXPHOS and FA metabolism of IFN-I treated wt and Irf9-/- BMDMs. (B) Extracted ion counts (EIC) of intracellular free FAs normalized to tubulin (n = 3 per condition), (C) mRNA expression of Cd36 (n = 3 per condition), (E) OCR and (F) ECAR in BMDMs 6h post-infection or medium treatment. (D) Three replicates of Western blots used to quantify ACC phosphorylation in BMDMs 6h post-infection. (TIF) [file ppat.1009697.s002.tif]

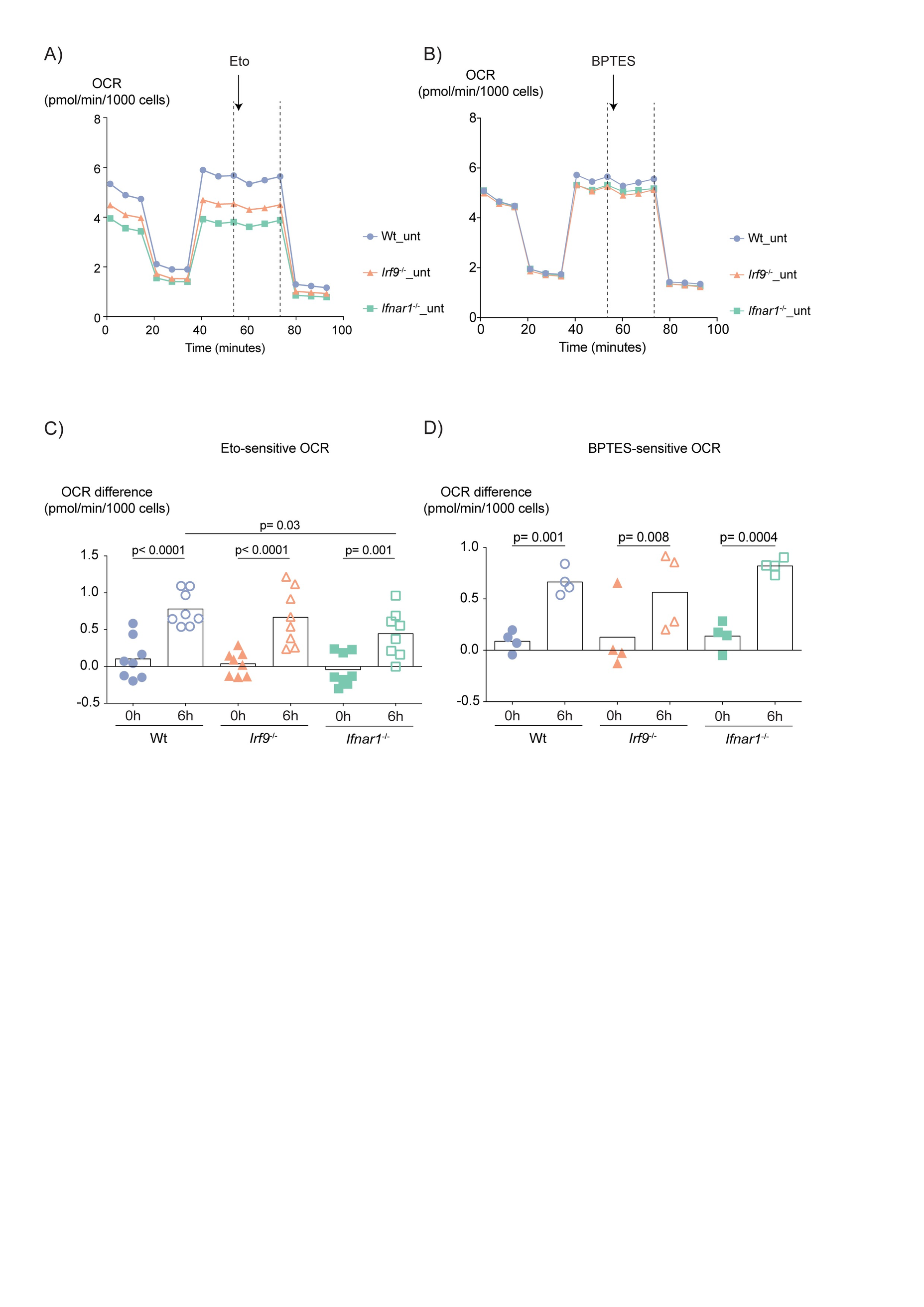

Supplement: S3 Fig — Mean OCR of four replicates of (A) Etomoxir- and (B) BPTES-treated uninfected BMDMs that are shown in Fig 2. The inhibitors were added at the time point indicated by the arrow. OCR difference of (C) Etomoxir-treated and (D) BPTES-treated BMDMs calculated by subtracting OCR at 73 min (addition of Rotenone/Antimycin) from OCR at 53 min. OCR differences were calculated using OCR values measured at time points indicated with dashed lines. Bars show the mean values. P values were calculated using ANOVA corrected for multiple testing with Dunnett’s post-hoc test. (TIF) [file ppat.1009697.s003.tif]

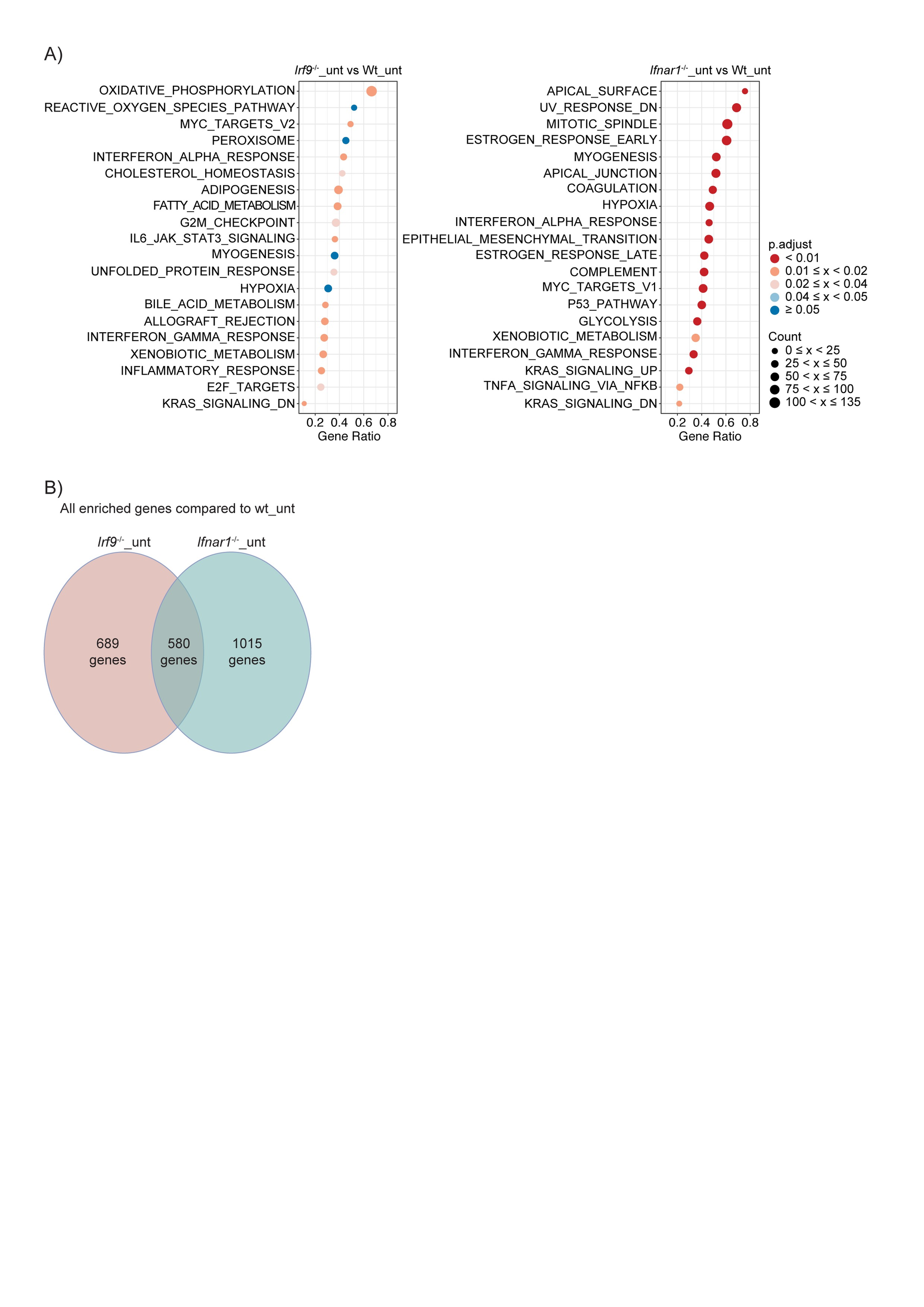

Supplement: S4 Fig — (A) Dot plots of GSEA results showing first 20 enriched pathways in uninfected (A) Irf9-/- and Ifnar1-/- compared to uninfected wt. Color and size of the dots represent adjusted p values and the number of the genes represented from a certain gene set, respectively. The x-axes were calculated by the sum of the core-enriched genes divided by its set size and ordered decreasingly. The y-axis represents the corresponding gene set name. (B) Venn diagram showing the number of genes enriched in uninfected Irf9-/- and Ifnar1-/- compared to uninfected wt. (TIF) [file ppat.1009697.s004.tif]

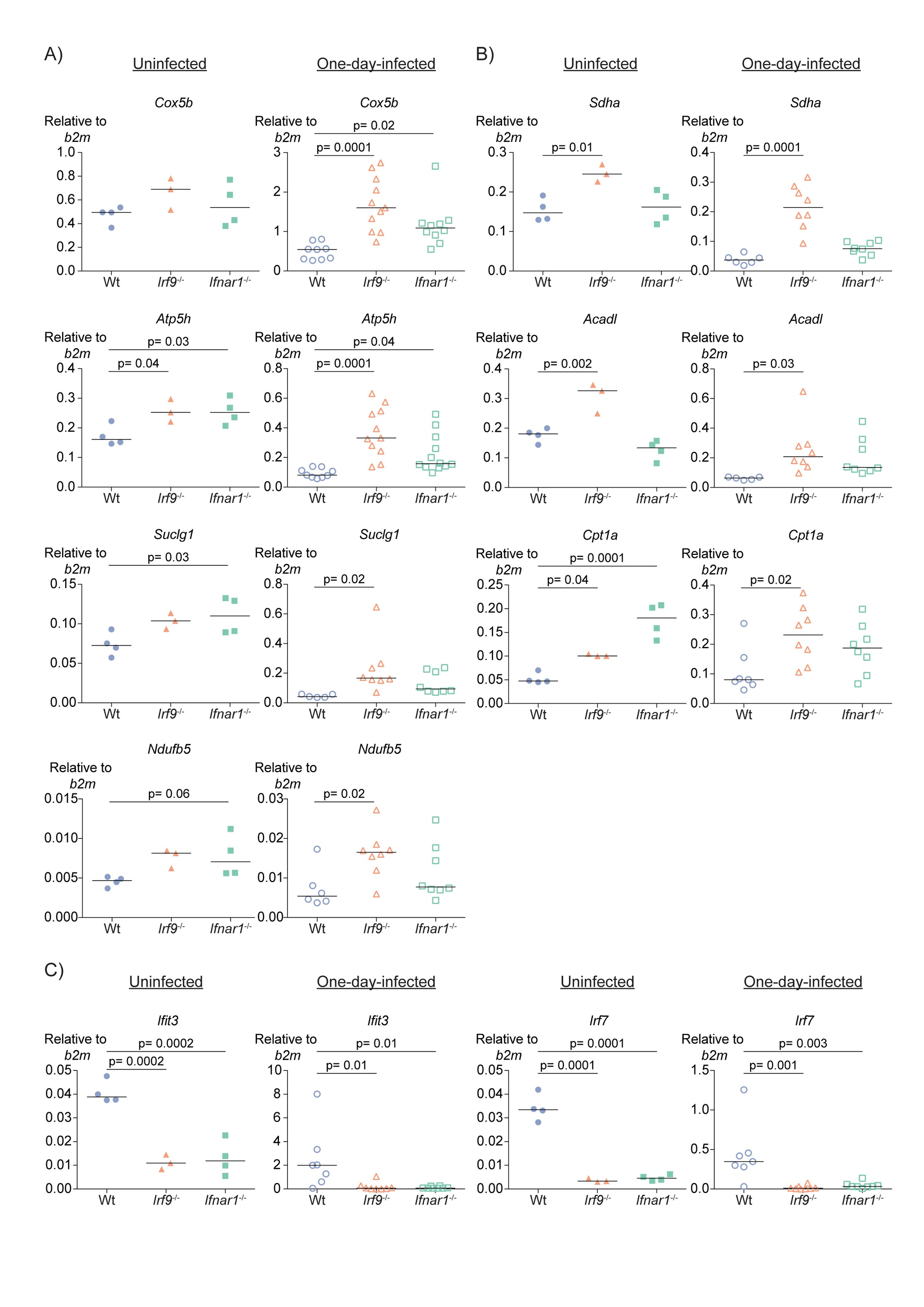

Supplement: S5 Fig — Quantitative RT-PCR of (A) OXPHOS, (B) FA metabolism and (C) IFN-stimulated genes from uninfected and one-day infected mouse livers. P values were calculated using ANOVA corrected for multiple testing with Dunnett’s post-hoc test. (TIF) [file ppat.1009697.s005.tif]

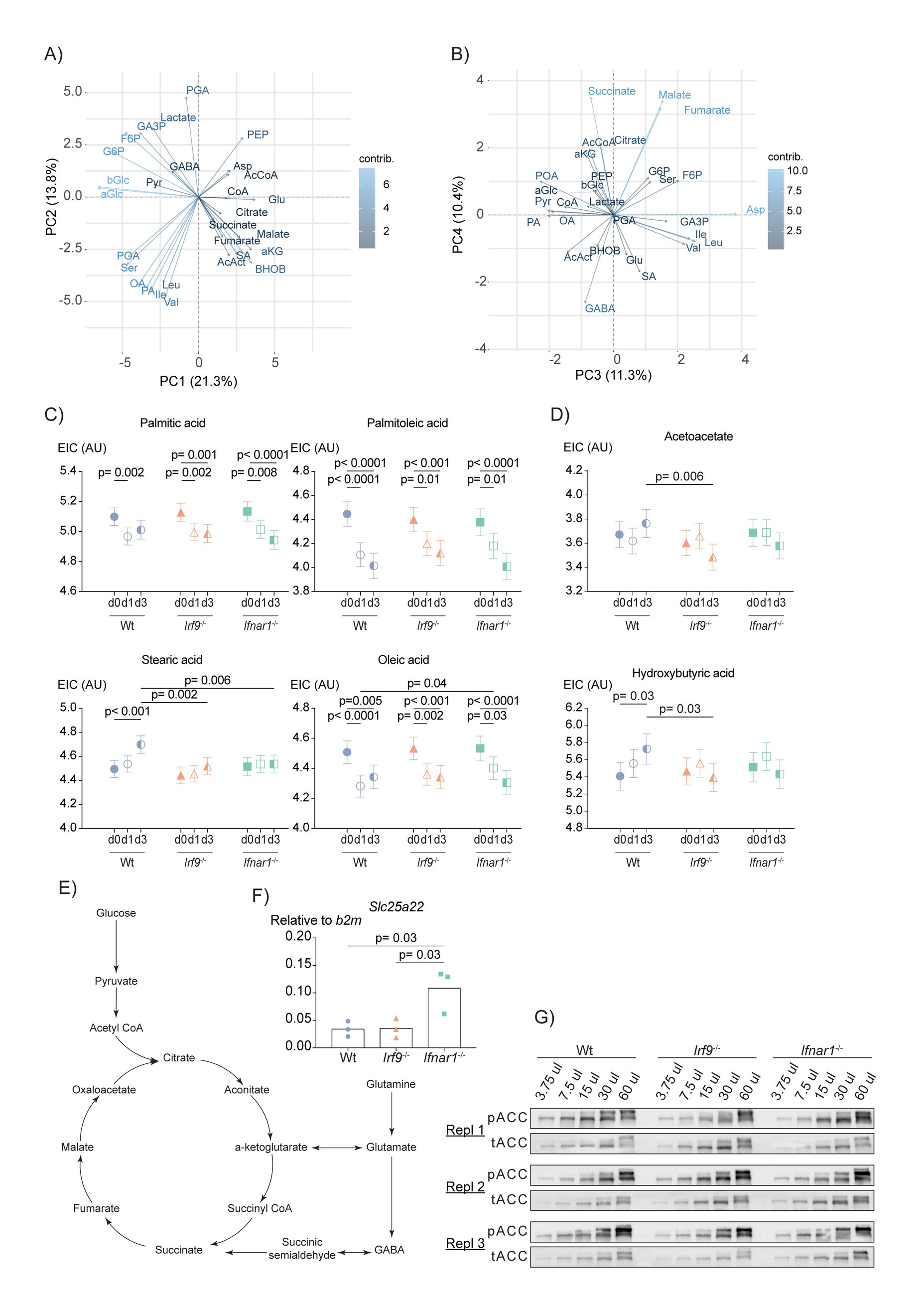

Supplement: S6 Fig — (A-B) Loading plots showing the contribution of each metabolite to clustering with vectors. Length and color of the vectors represent their contribution to separation of different clusters. Impact of each metabolite on a certain cluster separation is reflected in the direction of the vectors. Percentage of total variance is indicated in the axis label. The values are corrected for liver weight, genotype, sex of the mouse and time point effects. ANCOVA test was performed. Extracted ion counts (EIC) of intracellular (C) FAs and (D) ketone bodies in wt, Irf9-/- and Ifnar1-/- mice one- or three-days post-infection or PBS injection. False discovery rate p values were calculated using values corrected for liver weight, genotype, sex of the mouse and time point effects. ANCOVA test was performed. (E) Scheme showing glutaminolysis replenishing the TCA cycle. (F) mRNA expression of glutamate carrier Slc25a22 in uninfected wt, Irf9-/- and Ifnar1-/- livers. P values were calculated using ANOVA with Tukey post-hoc test. (G) Three replicates of Western blots used to quantify ACC phosphorylation in livers one day post-infection. Contrib.: contribution, PC: Principal component, PGA: Phosphoglyceric acid, PEP: Phosphoenolpyruvate, Asp: Aspartic acid, AcCoA: Acetyl CoA, Glu: Glutamate, aKG: alpha-ketoglutarate, BHOB: Hydroxybutyric acid, SA: Stearic acid, AcAct: Acetoacetate, Val: Valine, Leu: Leucine, Ile: Isoleucine, PA: Palmitic acid, OA: oleic acid, Ser: Serine, POA: Palmitoleic acid, aGlc: alpha-D-glucose, bGlc: beta-D-glucose, Pyr: Pyruvate, G6P: Glucose-6-phosphate, GA3P: Glyceraldehyde-3-phosphate. (TIF) [file ppat.1009697.s006.tif]
