# Supplementary material for: Listeria monocytogenes infection rewires host metabolism with regulatory input from type I interferons
Source: PLoS Pathog. 2021 Jul 8;17(7):e1009697. doi: 10.1371/journal.ppat.1009697 (PMC8266069; doi:10.1371/journal.ppat.1009697)
Supplement: Attachment — Submitted filename: Point-by-point reply to PP reviewers.pdf [file ppat.1009697.s014.pdf]

Point-by-point reply to the reviewer's comments.

We thank all reviewers for their time to scrutinize our manuscript and help us to improve it with their comments.

Reviewer 1

Major Points:

1. The puzzling choice of IP injection is all the more surprising since the authors have previously demonstrated that IFN-I have opposite roles in mice infected by the IP route or by the natural, more relevant oral route. Are the different contributions to infection control of IRF9 and IFNAR also seen in mice orally infected with the LO28InlA* strain?

Our aim was to identify factors contributing to the protective effects of the *Ifnar1*$^{-/-}$ and whether these are regulated through its transcriptional effector IRF9. Since the protective effects of the Ifnar1 ko are not seen after intragastric infection we chose the i.p. route. While the intragastric route can be considered to better resemble the natural infection route, the i.p. model allows one to study detrimental effects of type I IFN that have been noted with several bacterial pathogens including Mycobacteria in humans.

2. The study would greatly benefit from an in vivo experiment showing the role of some of the identified metabolites in the susceptibility of these mice to infection.

We have performed an experiment now shown as Figure 5E (see also text, lines 314-321), demonstrating that inhibition of fatty acid oxidation indeed reduces the bacterial burden in spleen and liver in wt, but not *Ifnar1*$^{-/-}$ or *Irf9*$^{-/-}$ mice. This allows us to conclude that reduced fatty acid oxidation contributes to the protective effect of IFNAR deficiency that is well established in the literature. We have also injected mice with GABA, however, without a detectable effect on bacterial loads of the *Listeria monocytogenes (L. monocytogenes)* target organs (Additional Figure 1_Additional figure 1 text).

3. CFU/well (instead of fluorescence) should be determined at 2, 6 and 24 hours (if cell viability is not affected). A time "zero" control should be added to demonstrate that the capacity of internalization is similar in WT, Ifr9-/- and Ifnar1-/- cells.

We have replaced the original flow-cytometry-based assay with a more conventional Gentamicin protection/CFU assay under conditions applied successfully in our and other labs (https://doi.org/10.1111/j.1462-5822.2007.01114.x,
DOI:https://doi.org/10.4049/jimmunol.0900460; DOI: 10.1084/jem.20040976 ; Fig. 1H). We titrate every new batch of gentamicin for a concentration killing extracellular bacteria, but allowing for growth of intracellular bacteria.
In this assay the slight decrease of bacteria in *Ifnar1*$^{-/-}$ and *Irf9*$^{-/-}$ macrophages was not observed, in line with previous reports (DOI: 10.1084/jem.20040976). A difference between this and the original approach is that the flow cytometry-based assay includes signals stemming from dead bacteria. For this reason, we excluded the original figure and show the gentamicin protection assay as suggested.

Minor Issues:

1. At day 3, injection of 10e6 LO28 led to death of some WT and Ifnar1-/- mice, and thus heterogeneity. It could be worth measuring bacterial survival upon infection with a sublethal inoculum.

The reviewer is correct in pointing out a different fraction of mice succumbing to infection after day 3. However, our priority was to replicate the exact conditions of the survival assay which cannot be performed with a lower inoculum, due to a too low number of wt mice that would be killed by infection. However, we are convinced that, given the large number of experimental samples in the organ load assays (n>10), it is highly unlikely that a few additional values, even if extreme, would spread the means such that they explain the vast differences in survival, particularly between wt and *Irf9*$^{-/-}$. Therefore, we think the statement that the genotype effects have a resilience in addition to a resistance component is justified.

2. Many phenotypes are investigated in WT, Ifr9-/- and Ifnar1-/- backgrounds with or without infection. A summary of what is IRF9-dependent, IFNAR-dependent, IRF9- and IFNAR-dependent and IRF9- and IFNAR-independent would be helpful in the discussion. For instance, figure 6 could be completed with an Ifr9$^{-/-}$ cell, liver data and uninfected data

We point out important differences between the genotypes in several sections of the discussion (Lines 350-355, 366-367, 385-386, 390-392, 397-406, 413, 422-425). In addition, we have revised Fig. 6 to display the most important outcome of our findings also with respect to the differences between *Irf9*$^{-/-}$ and *Ifnar1*$^{-/-}$ as suggested by the referee. More differences have emerged, e.g. in the GSEA or the analysis of CCL2 levels, but we cannot interpret how they contribute to the distinct phenotypes.

3. Line 159 It is not clear why the Wang reference is introduced here.

An error which has been corrected. Thank you for pointing it out.

4-Line 396 The mice genotype should be indicated.

Mouse genotypes and genetic background have been added in materials and methods (Line 433).

5-Fig S1C is not introduced.

The Fig. is now introduced, line 130.

6-Some references should be completed (journal, page numbers etc.).

We have corrected this.

7-Western blots should be shown (Figures 2 and 5)

These are now shown in Supp figs 2d and 6g.

8-It would help to indicate clearly on figures what is related to uninfected or infected data

We have added source and infection to the relevant figures.

9-The unacceptable quality of the main figures, probably due to file conversion, should be improved. Figures 3 and 4 are partially illegible...

We apologize, this must result from the automatic pdf conversion during submission. The originals have excellent resolution.

Reviewer 2

Major Points:

1) The observation that IRF9 mice are even more resistant than IFNAR mice is interesting and novel but there is no work presented to explain this phenomenon. As the transcriptomics data in figure 3 focus only on potential metabolic differences which appear to not explain the phenotype, significant additional studies are necessary to dissect the reason for this phenotype.

The reviewer is correct in pointing out that our manuscript provides no satisfying explanation for the differences between *Ifnar1*$^{-/-}$ and *Irf9*$^{-/-}$ in withstanding *L. monocytogenes* infection. It was our choice to base the manuscript entirely on the difference between wt and *Ifnar1*$^{-/-}$. However, we decided against this option as the manuscript does contain data showing parameters of infection that might have been, but ended up NOT being responsible for the difference. This will help us and others in designing follow-up experiments. Furthermore, the RNAseq data show a significant number of differentially induced genes which is surprising given the prominent role of Jak-Stat signaling and the ISGF3 complex in the transcriptional response to IFN. To our knowledge this is a novel observation.

2) Although not a major contributor to the story the authors try to tell, the cell intrinsic differences in macrophage control of Listeria infection presented in figure 1H are inconsistent with the existing literature Figure 1H_ Lines 168-172) that has found no cell intrinsic difference in IFNAR macrophage control of Listeria infection (ex. Auerbuch et al 2004). Why the authors see something different is not explained and this is important as these same cells are then used for all the metabolic analysis in figure 2 and it is critical to know if there is something different than other papers in the field.

We have replaced the original flow-cytometry-based assay with the more conventional Gentamicin protection/CFU assay under conditions applied successfully in our and other labs
In this assay the slight decrease of bacteria in *Ifnar1*$^{-/-}$ and *Irf9*$^{-/-}$ macrophages was not observed, in line with previous reports (DOI: 10.1084/jem.20040976). A difference between this and the original approach is that the flow cytometry-based assay includes signals stemming from dead bacteria. For this reason, we excluded the original figure and show the gentamicin protection assay (Figure 1H, Lines 146-150).

3) The transcriptomics data are presented in a very confusing way and no validation of any of the data were performed (and comments in Summary).

We tried to improve the display of the data to make it clearer by including heatmaps. We have also validated some of the genes and show this in supplementary figure 5. The referee is correct however that there is no simple relationship between the behavior of mRNAs of metabolism genes and the changes in metabolism according to metabolomics. We do not always understand this relationship, but have to accept it as a fact. We discuss potential reasons underlying this non-linear causality (Lines 230-237, 406-412).
Importantly, the GSEA allows us to consider metabolism as a facet contributing to the impact of IFN on *L. monocytogenes* infection. All conclusions how this might be related are based on the

metabolomics, which is a more downstream analysis, not the behavior of metabolic mRNAs which we often cannot interpret.

4) Figure 4 suffers the same readability issues…as presented it is difficult to glean meaningful information from the data, as the authors themselves seem to concede in stating simply that there are differences in metabolites. This data is at least followed up with analysis of specific metabolites in figure 5.

It is indeed a challenge to present the large data sets and their statistical analysis in an intuitive figure. We have tried to do better in the revised manuscript by separating the PCA of the metabolomics data and the vectors showing the contribution of those metabolites to the clustering of uninfected and infected mice or of the different genotypes in the PCA. In order not to lose the quantitative information about the contribution of metabolites in the main figure, the bar graphs in figs. 4E and F document the quantitative contribution of metabolites to the differences caused by infection and the differences caused by the genotypes.

5) Most importantly, there is. In the end the manuscript ends up as a list of data with no appreciation for the biological impact of any of it. The authors themselves state "the rather small metabolic effects observed in our macrophage experiments alone are unlikely to make major contributions to the substantial differences between Irf9-/- and Ifnar1-/- mice in the resistance to L. monocytogenes infection". In the end, this is the crux of the issue, data was presented that there are metabolic differences in the knockout mice but the phenotypes are small and there is no indication that they are biologically relevant without additional experimentation to manipulate the pathways and assess subsequent Listeria infection outcomes.

To overcome this, we have performed an experiment now shown as Figure 5E (see also text, Lines 314-321), demonstrating that inhibition of fatty acid oxidation, the main implication of the metabolism data, reduces the bacterial burden in spleen and liver in wt, but not *Ifnar1*$^{-/-}$ or *Irf9*$^{-/-}$ mice. This allows us to conclude that reduced fatty acid oxidation contributes to the protective effect of IFNAR deficiency that is well established in the literature. We have also injected mice with GABA, however, without a detectable effect on bacterial loads of organs (Additional Figure 1_Additional figure 1 text).

Reviewer 3

Major Points:

1. Given the varied metabolic contribution of macrophages/circulating monocytes compared with hepatocytes do the authors hypothesize that the same pathways are at play in both conditions? The final conclusions seem to indicate that they suspect the same pathway but it seems like the RNA-seq data would have incorporated a mix of macrophages and hepatocytes whereas the BMDM data would represent metabolic data solely derived from immune cells

We have been careful not to overinterpret our data with regard to similarities or differences between hepatocytes and macrophages. A common denominator according to our data is depicted in Fig. 6. 70% of cells and 80% of the volume of the liver are due to hepatocytes. Therefore, an RNA-seq experiment should predominantly represent this cell type, based on the predominance of reads derived from hepatocyte mRNA.

2. When the authors mined their previous BMDM data from Platanitis E, et al., how much does it overlap with the RNA-seq from this analysis? Do they find the same proteins up or down?

We provide additional figure 3 showing this overlap for the reviewer's information.

3. Along these lines could they infect animals and separate out immune cells from hepatocytes and analyze levels of p-ACC or GABA to access whether the immune cells and hepatocytes have the same response?

This is an excellent suggestion. Unfortunately, the dissociation and separation of individual liver cell types is not an established technology in our lab. It would require a considerable amount of time to get this done, particularly because we would have to compare infected and uninfected animals with considerable differences in the composition of the nonparenchymal cell fraction.

4. They could also add infection of primary hepatocytes or a hepatocyte cell line to sort this out

This poses the same problem as above, it would require us to isolate hepatocytes from wt and *Ifnar1*$^{-/-}$ mice as we do not have an *Ifnar1*$^{-/-}$ deficient hepatocyte line.

5. If the authors expect both immune cells and hepatocytes to act in the same manner, this should just be more fully discussed throughout the text and highlighted on the figures where the source material comes from, while the figure legends and text indicate the source material, it would be better to see it on the figures as well.

We added a paragraph in the discussion to address the potential relationships between macrophage and hepatocyte metabolism (Lines 373-377, 420-422). For the reasons given above, there are clear limits of interpretation with the available data. The figures now indicate the source material as suggested by the reviewer.

6. Adoptive transfer experiments or tissue specific deletion (hepatocyte versus immune CRE) could address this issue elegantly but could be reserved for future work.

We performed adoptive transfer experiments in accordance with the reviewer's suggestion. Within the time period available for revision, we could not wait very long before analyzing the reconstituted mice, which may somewhat reduce the contribution of long-lived cells. Thus, the data are preliminary and only provided for the reviewer's information (Additional figure 2). Nevertheless, they do quite clearly indicate a contribution of immune cells to the *Ifnar1*$^{-/-}$ phenotype and a smaller role of *Ifnar1*$^{-/-}$ nonhematopoietic cells. This is in accordance with hepatocyte metabolism making a contribution to the IFN-dependent increase in susceptibility against *L. monocytogenes* infection.

7. The authors spend a fair amount of time in the introduction explaining the role of various metabolic pathways in activated macrophages. Are BMDMs the best model to study this phenomenon or would resident or peritoneal macrophages be a better representation of the monocytes that they are interested in in vivo? How would polarization or pretreatment to activate BMDMs affect or change the phenotype

We have analyzed resident peritoneal macrophages as suggested and observed that these cells show a significant difference to BMDM (Additional figure 4). The impact of infection on OCR and ECAR is rather small and ACC1 phosphorylation in *Ifnar1*$^{-/-}$ cells is increased, rather than decreased. This observation is in line with peritoneal macrophages having less M1 polarity than BMDM. Since the defense against *L. monocytogenes* in the peritoneum is dominated by M1-polarized inflammatory monocytes BMDM may in fact be a better model to study metabolic effects. In support of this interpretation, the new in-vivo data with FAO inhibitor (Fig. 5E) show that our notion of fatty acid oxidation contributing to the susceptibility against *L. monocytogenes* and its decrease contributing to the resistance increase of *Ifnar1*$^{-/-}$ cells is correct.

8. The authors should upload their RNA-seq data to a searchable database or upload the log transformed data in the form of an excel table that highlights the individual proteins detected and their values. They have done this with the enrichment analyses but unless I missed it the complete RNA-seq analysis is not part of the supplementary data.

All the RNA sequencing data is available in NCBI GEO repository on series record GSE 162448. This sentence has been added to materials and methods (Line 494)

9. The authors did not show the raw Western blot data for phospho ACC levels. At least a representative blot should be shown, but it would be preferable to see all three in supplementary data in line with increased transparency.

The data have been added, as suggested by the reviewer (Supplementary figure 2D and Supplementary figure 6G).

Discussion points:

1. In their previous work the authors often compared IRF3/7 KO animals to IFNAR. It is beyond the scope of this paper to cross compare those cells/animals but I wonder if they would discuss what they would expect? Would cytosolic DNA/RNA signaling that is IRF9-independent potentially be responsible for this phenotype observed in the IFNAR KO? Along these lines, when they cross compare IFNAR and IRF9 KO mice they have overlapping genes in Venn diagrams but I was not able to find the list of these genes. Are they in a table that I missed?

We surmise that IRF3/7 dko mice would resemble the *Ifnar1*$^{-/-}$. However, after the experience with the IRF9 ko we have become very aware of the possibility that IRFs lead an IFN-independent life. In fact, there is some literature suggesting just that, interestingly with a metabolism connection. Nucleic acid signaling has been described in so many situations, immunological and non-immunological. However, I would not be able to explain why and how it complements IFNAR1 and IRF9 deficiencies differently. My favorite speculation is that Stat-independent signaling by the IFNAR makes the difference. We apologize for the omission of a table with genes differentially expressed in *Ifnar1*$^{-/-}$ and *Irf9*$^{-/-}$. Supporting table 6 with this information has now been added.

For the PCA analysis, I think the figure should be revised so one can see the general distribution of the phenotypes and the individual differences in pathways that they wish to highlight could be displayed as bar graphs below. As it stands, it is very difficult to make out the various conditions due to the labeling.

It is indeed a challenge to present the large data sets and their statistical analysis in an intuitive figure. We have tried to do better in the revised manuscript by separating the PCA of the metabolomics data and the vectors showing the contribution of those metabolites to the clustering of uninfected and infected or mice or of the different genotypes in the PCA. In order not to lose the quantitative information about the contribution of metabolites in the main figure, the bar graphs in figs. 4E and F document the quantitative contribution of metabolites to the differences caused by infection and the differences caused by the genotypes.

The authors have included diagrams of the metabolic pathways that they discuss in the supplementary figures but it may be worthwhile to incorporate such diagrams into the figures so that readers can cross compare

We apologize for not having come up with a good idea to do this without overloading the figures.

A)

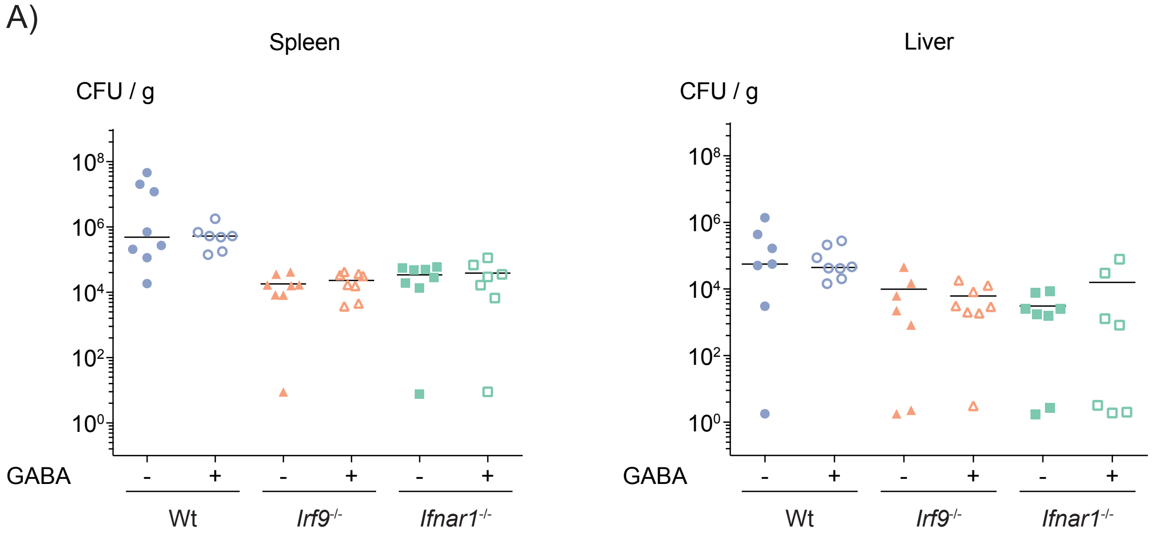

Additional Figure 1: Relevance of GABA during *in vivo L. monocytogenes* infection
Our experiments showed that Ifnar1-/- mice have increased levels of GABA before and after infection. As per the suggestion of the reviewers, we injected mice with 200mg/kg GABA or corresponding volume of PBS daily from the day of infection onwards [1]. Then we harvested the spleens and livers of infected mice on the third day of infection and performed CFU experiments.

Our experiment showed no effect of GABA in protection from *L. monocytogenes* infection. These data suggest that although GABAergic signaling has a role in guarding mice against the adverse impact of *L. monocytogenes* infection [1], GABA is solely indirectly involved in this.

Additional Figure 2: Bacterial loads of bone-marrow-transplanted mice

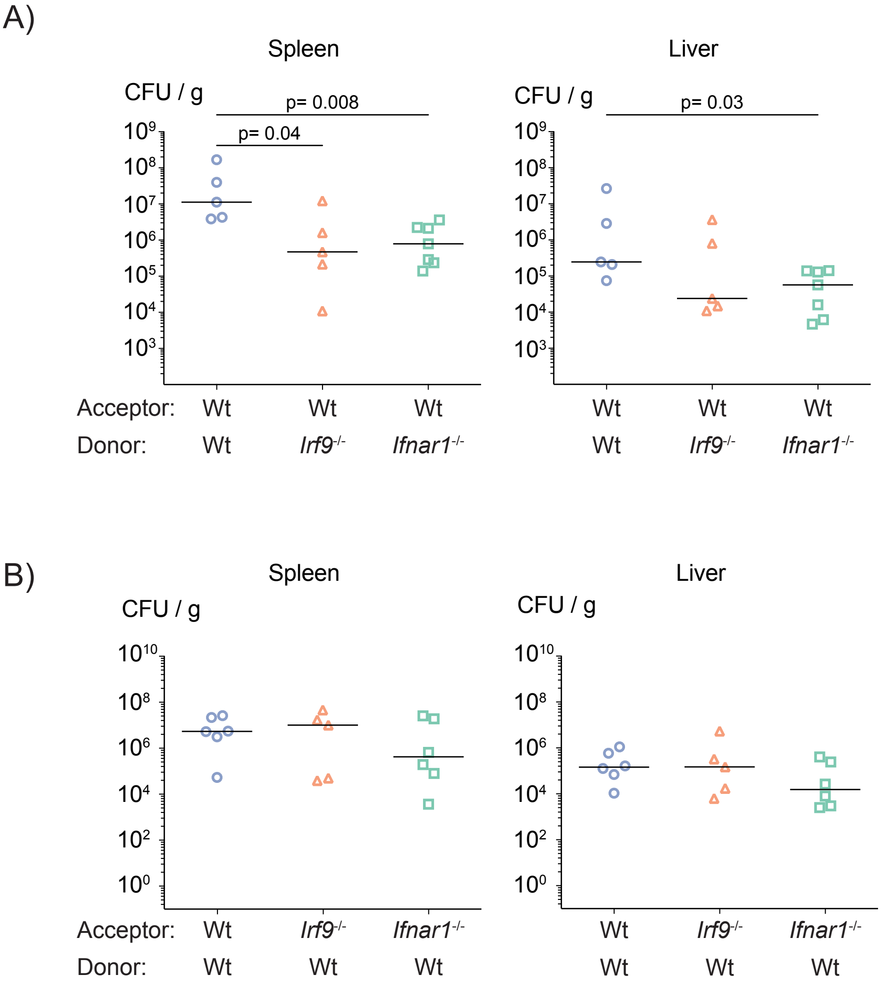

Additional Figure 2: Bacterial loads of bone-marrow-transplanted mice

In order to find out the source of protection against Lm infection in the Irf9-/- and Ifnar1-/- mice, we lethally irradiated the CD45.1+ wt mice and transplanted them with bone marrow cells (BM) from CD45.2+ wt, Irf9-/- or Ifnar1-/- mice. Three weeks post infection, we infected the chimeras with L. monocytogenes for two days and detected lower bacterial load in the spleen and liver of mice with Irf9-/- or Ifnar1-/- BM than wt BM transplanted mice (Additional Fig 2A). On the other hand, when we transplanted CD45.1+ wt BM into CD45.2+ wt, Irf9-/- or Ifnar1-/- mice, we did not measure any change in the bacterial burdens of Irf9-/- in comparison to wt transplanted mice but a lower trend of in the Ifnar1-/- mice pointing to a role of non-hematopoietic cells in protection against *L. monocytogenes* (Additional Fig 2B). From these results we can conclude that in the early phase of infection, mainly immune cells are responsible from immunity against *L. monocytogenes*. Our preliminary data suggest that in Ifnar1-/- mice non-hematopoietic cells might also play a role. However, due to time constraints and mouse availability in the COVID times, we could not address metabolic questions in this context.

Additional Figure 3: BMDM and Liver RNAseq comparison

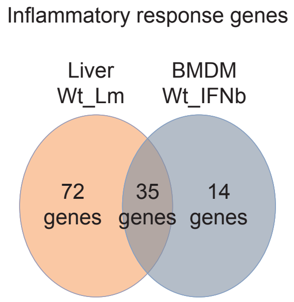

	Inflammatory response genes	
**Enriched in wt_Lm liver**	**Common to both**	**Enriched in wt_IFN-I BMDMs**
Cxcl10	Mefv	Il7r
Slc28a2	Il1a	Atp2a2
Tlr2	Il4ra	Fzd5
Irf7	Cybb	Aqp9
Eif2ak2	Ffar2	Edn1
Il10ra	Tnfsf15	Il10
Tlr1	Nmi	Il18
Lcp2	Btg2	Calcrl
Tnfrsf9	Nfkb1	Ahr
Icam1	Il18rap	Tnfrsf1b
Serpine1	Ptpre	Sphk1
Ripk2	Hbegf	Itgb8
Ccrl2	Lpar1	F3
Bst2	Cd69	Lck
Nampt	Olr1	
Csf3r	Ccl7	
Tlr3	Cxcl9	
Osmr	Ly6e	
Nlrp3	Csf1	
Cmklr1	Tapbp	
Ptafr	Slc11a2	
Marco	Cxcl11	
Slc7a1	Slc31a1	
Il15	Tnfaip6	
Mxd1	Sell	
Plaur	Slc31a2	
Osm	Rasgrp1	
Clec5a	Irf1	
Irak2	Ifngr2	
Msr1	Gna15	
Cxcr6	Tnfsf10	
Klf6	Nod2	
Il2rb	Il6	
Fpr1	Slamf1	
Ccl5	Ccl12	
Axl		
Il18r1		
Ptgir		
Cd48		
Nfkbia		
Ccl24		
Ifitm1		
Cxcl5		
Sema4d		
Cd40		
Dcbld2		

Additional Figure 3: Comparison of RNA sequencing data from IFN-I-treated BMDM and *L. monocytogenes*-infected mouse livers

We compared the RNAseq data from our previous publication performed in IFN-I-treated wt BMDMs [2] with the RNAseq data of *L. monocytogenes*-infected wt mouse livers from our current study. Gene set enrichment analysis of the datasets showed inflammatory response gene set among the top enriched pathways in IFN-I-treated wt BMDMs and infected wt livers. Comparison of the list of these genes that are significantly upregulated upon treatment or infection showed 72 and 14 genes were exclusively enriched in liver and BMDMs, respectively, while 35 genes are shared between the two. The list of these genes can be seen in the table.

Additional Figure 4: Metabolic responses of *ex vivo* infected peritoneal macrophages

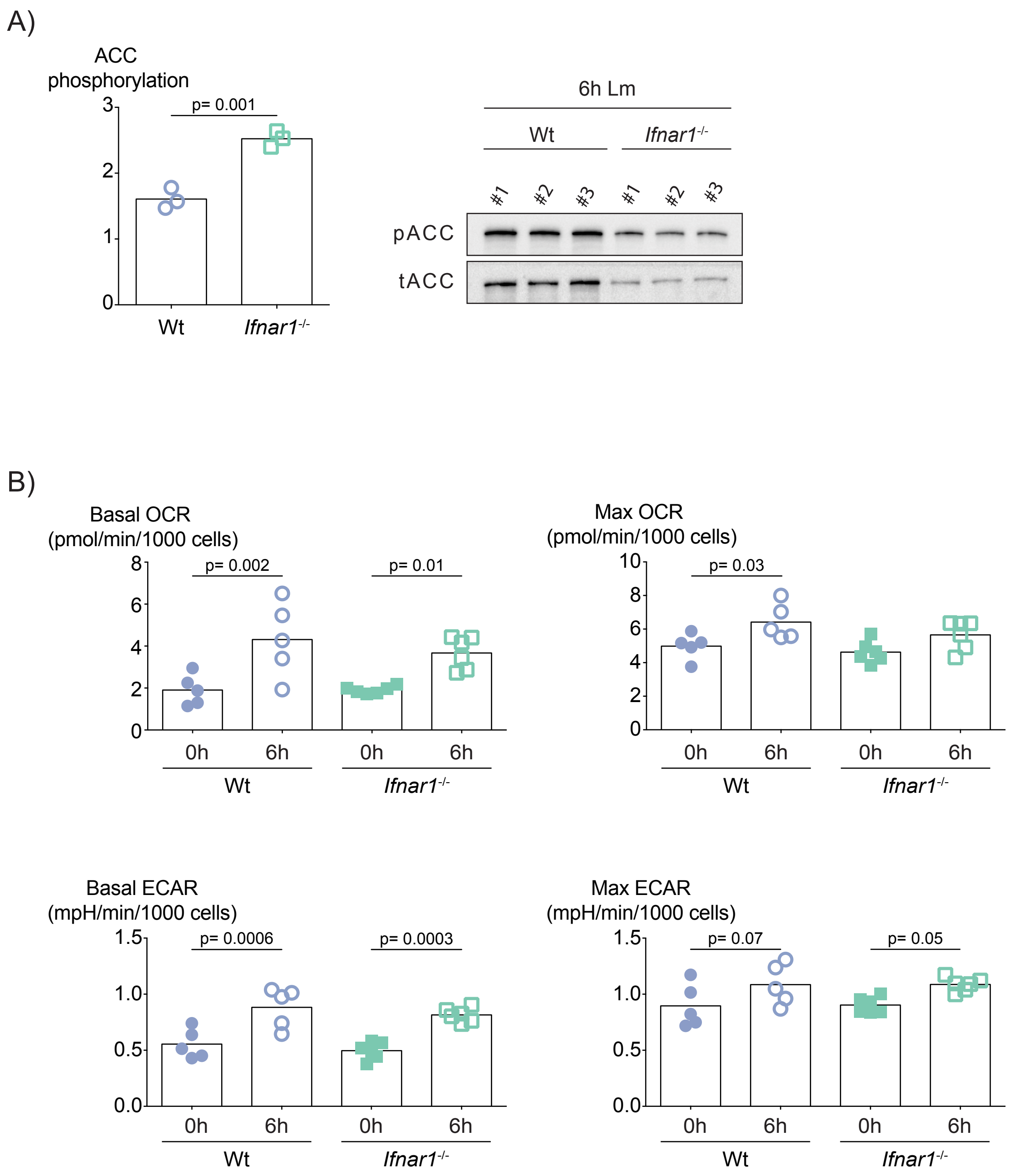

Additional Figure 4: Metabolic responses of *ex vivo* infected peritoneal macrophages

Peritoneal cells were harvested in 10% FCS + PBS and 5.3*10^5 and 3*10^6 cells were seeded on 96-well Seahorse and 6-well plates. 30% of the total peritoneal cell population was assumed to be macrophages. The non-adherent cells were washed away after 2 h of incubation and the cells were infected with MOI 10 *L. monocytogenes*. Seahorse and western blotting were performed as explained in the manuscript.

Our data show that in contrast to BMDMs, Ifnar1-/- peritoneal macrophages display higher ACC phosphorylation than wt peritoneal macrophages (Additional Fig 4A). Seahorse measurement of OCR and ECAR showed an induction for both OXPHOS and glycolysis after infection (Additional Fig 4B). However, we did not detect an impact of genotype which could derive from the fact that BMDMs are skewed to M1 macrophage direction than peritoneal macrophages [3].

REFERENCES

1. Kim JK, Kim YS, Lee H-M, Jin HS, Neupane C, Kim S, et al. GABAergic signaling linked to autophagy enhances host protection against intracellular bacterial infections. Nat Commun. 2018;9: 4184. doi:10.1038/s41467-018-06487-5

2. Platanitis E, Demiroz D, Schneller A, Fischer K, Capelle C, Hartl M, et al. A molecular switch from STAT2-IRF9 to ISGF3 underlies interferon-induced gene transcription. Nat Commun. 2019;10: 2921. doi:10.1038/s41467-019-10970-y

3. Zajd CM, Ziemba AM, Miralles GM, Nguyen T, Feustel PJ, Dunn SM, et al. Bone Marrow-Derived and Elicited Peritoneal Macrophages Are Not Created Equal: The Questions Asked Dictate the Cell Type Used. Front Immunol. 2020;11: 269. doi:10.3389/fimmu.2020.00269
